# ‘Making of a Strong Woman’: a constructivist grounded theory of the experiences of young women around menarche in Papua New Guinea

**DOI:** 10.1186/s12905-021-01229-0

**Published:** 2021-04-08

**Authors:** Elizabeth Maulingin-Gumbaketi, Sarah Larkins, Ronny Gunnarsson, Gun Rembeck, Maxine Whittaker, Michelle Redman-MacLaren

**Affiliations:** 1 College of Medicine and Dentistry, James Cook University, Townsville, Australia; 2 College of Public Health, Medical and Veterinary Sciences, James Cook University, Townsville, Australia; 3 Research and Development Primary Health Care, Research and Development Center Södra Älvsborg, Region Vastra Gotland, Sweden; 4 Primary Health Care, School of Public Health and Community Medicine, Institute of Medicine, Sahlgrenska Academy, University of Gothenburg, Gothenburg, Sweden; 5 Regionhälsan, Borås Youth Health Center, Borås, Sweden

**Keywords:** Menarche, Menstrual health and hygiene, Reproductive health, Papua New Guinea

## Abstract

**Background:**

Menarche, the first menstruation, is a significant developmental milestone for females. In Papua New Guinea (PNG), menarche is an important socio-cultural event marking transition from girlhood to womanhood. PNG is a culturally and linguistically diverse nation, with wide-ranging socio-cultural beliefs and practices around menarche. This study explored post-menarcheal women’s understanding about body changes and menarche, preparation for menarche, and related cultural beliefs and practices at menarche.

**Methods:**

A constructivist grounded theory study was conducted with 98 female participants who originated from four PNG provinces: Eastern Highlands Province; East Sepik Province; Milne Bay Province; and National Capital District. The participants were purposively and theoretically sampled, with 10 focus group discussions and six individual interviews conducted using a semi-structured interview guide for data collection. Focus group discussions and interviews were voice recorded and transcribed. Data were inductively analyzed using initial, intermediate and advanced coding, memos and constant comparative methods to develop a theoretical model that explains women’s experiences at menarche. Interview participants also identified actions required to improve future experiences of girls at menarche in PNG.

**Results:**

A grounded theory comprising the core category of ‘Making of a Strong Woman’ and four interconnecting categories (‘Having Baby Sense’; ‘Beginning of Learning’; ‘Intensifying Learning’; and ‘Achieving Womanhood’) was constructed. ‘Urban’ and ‘Rural’ represented both geographical and socio-cultural intervening conditions that influence the experiences of girls at menarche. Experiences of young women at menarche were rooted in socio-cultural beliefs and practices. Women reported being physically and emotionally distressed and unprepared at onset of menarche. Mothers were considered important support, however, their ability to adequately prepare their daughters is limited by shame and secrecy. Despite these limitations, cultural practices at menarche provided an opportunity for intensive preparation of girls for womanhood.

**Conclusion:**

Limited pre-menarcheal awareness of the meaning of body changes and menarche of girls was linked to culture of shame and secrecy about open discussion on sexuality. However, traditional cultural practices provide an opportunity for collective support and focused learning for girls. Findings from this study have implications for broader sexual and reproductive health education programs in addressing menstrual health and hygiene in PNG, and the Pacific.

**Supplementary Information:**

The online version contains supplementary material available at 10.1186/s12905-021-01229-0.

## Background

Menarche (onset of first menstruation) is an important developmental milestone for adolescent females. Pre-menarche refers to six months before menarche and post-menarche is six months after menarche [1]. Both concepts will be used throughout the text.

Menarche marks reproductive maturation, and transition from girlhood to womanhood. Evidence from both Low and Middle Income countries (LMIC) [2], and High Income Countries (HIC) [3, 4] shows significant variation in the experiences of adolescent girls before and at menarche in relation to knowledge (meaning of body changes and menarche), attitudes and practices. These experiences are largely determined by local environmental factors such as socio-cultural beliefs and practices around menarche and menstruation [5–7].

Menarche is an important traditional social and cultural event in many areas of Papua New Guinea (PNG) [8]. Menarche signifies the ending of childhood and beginning of womanhood and is associated with cultural beliefs and ritualized practices. These beliefs and practices vary according to different cultural and language groups in PNG [9]. Despite social and cultural transitions due to colonization and globalization, certain aspects of these beliefs and practices (isolation, initiation, cleansing ceremonies and celebrations) are still observed in both rural and urban areas of PNG [10].

Papua New Guinea is one the most culturally diverse countries among the Pacific Island Countries and Territories (PICTs) with over 800 different cultural and language groups in the population of approximately eight million people [11, 12]. Social and cultural norms, beliefs and practices continue to regulate the lives of people in PNG. This is especially so for the approximately 80% of the population who still live in rural and remote areas [13]. However, these beliefs and practices are diminishing due to external influences such as introduced religion, colonization, globalization, urbanization and inter-marriage [9, 14, 15]. With increasing levels of urban migration, people’s lives are becoming less regulated by traditional socio-cultural norms, beliefs and practices because of the shift away from the traditional lifestyle and increasing level of education. National Capital District (NCD), where Port Moresby (country’s capital) is located, is increasingly becoming the melting pot of traditional and modern lifestyles of PNG [16, 17]. In NCD, high rates of domestic and sexual violence [18], teenage pregnancies [19–21] and Sexually Transmitted Infections (STI) including Human Immunodeficiency Virus (HIV) infections [9] are linked to the breakdown of socio-cultural norms and fabrics of the society.

Women comprise almost half of the total population in PNG, yet they typically have lower social status than men and continue to live with many challenges [22]. The predominantly patriarchal cultures in PNG place women in a disadvantaged position in a number of domains [9, 12, 23]. For example, the maternal mortality ratios are shockingly high with an estimated rate of 215 maternal deaths per 100,000 live births in 2015 [23, 24]. Women continue to experience high rates of sexual violence [9] with 44 percent of women reporting they had been sexually abused in a 2008 study [74]. Seventeen percent of the total burden of sexual abuse involves girls between the ages 13 and 14 years [22]. In the education arena, literacy and educational attainment for women and girls continue to remain low, with 39% of females compared to 61% males, enrolled in secondary schools in 2015 [22]. Furthermore, women are underrepresented in national politics, thus are unable to influence national policies, while men continue to have power and dominate decision making [12, 23]. However, in some matriarchal societies as on Bougainville Island, woman play a key role. They brokered peace negotiations during the secessionist war between 1989 and 2000 and continue to enact their traditional leadership roles in decision making [25].

Descriptive anthropological studies on various cultures in PNG have documented significant cultural beliefs and practices around menarche and subsequent menstruation [8, 26–28]. These studies were usually conducted by non-Papua New Guineans reporting the diversity of cultural beliefs and practices around menarche and subsequent menstruation in PNG. However, there is a dearth of information on the experiences (knowledge, attitude and practices) of young adolescent girls at menarche in PNG. Mohammed and colleagues’ recent formative study investigated menstruation in three nations of the Pacific (PNG, Fiji and Solomon Islands) [5], reporting cultural beliefs and restrictive practices that impact on the Menstrual Hygiene Management (MHM) of young girls and women. However, the study had two limitations. First, the broader socio-cultural location of the study sites were unclear—naming of study sites for any socio-cultural studies in PNG, for example is important given its cultural hyper-diversity. Second, the study was descriptive in nature. Consequently, authors were unable to provide nuanced understanding of the socio-cultural phenomena around the restrictive practices that impact on MHM, critical for policy and programming on menstrual health and hygiene management as well as sexual and reproductive health more broadly in the Pacific context [29].

Deeper and more contextual understanding about the meaning of socio-cultural beliefs and practices and their implications for girls’ experiences is required given the vast and diverse cultures in and among different countries [2, 29]. The substantive area of inquiry for this constructivist grounded theory study was to explore the experiences of menarche with women and girls in PNG to inform strategies that support a positive transition to womanhood. Specifically, this grounded theory study sought to:

1. Understand social and cultural factors impacting on the experience of adolescent girls at menarche in PNG;
2. Understand how these experiences shape their knowledge, perceptions and practices at menarche;
3. Understand the perceived role for pre-menarcheal preparations of adolescent girls and the type of messages being taught; and
4. Identify the best solutions for local-level action on menstrual health and hygiene.

## Methods

Constructivist Grounded Theory (CGT) methodology was used to inductively generate a theory to explain both rural and urban women’s experience of menarche in PNG.

**Fig. 1:**
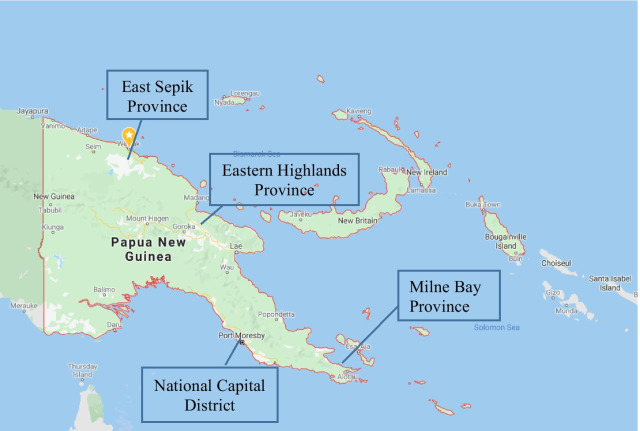
Map of Papua New Guinea showing study sites. *Source*: Google maps [62]

CGT was the methodology used as it is a useful methodology for exploring participants’ experience of menarche in their natural settings and enables the researcher to inductively co-create highly contextualized knowledge with participants [33]. In addition, CGT is consistent with the constructivist philosophical stance of the lead author [73]. Data collection was conducted in Port Moresby, National Capital District, PNG with post-menarcheal women from four provinces (Fig. 1): East Sepik Province (ESP); Milne Bay Province (MBP); Eastern Highlands Province (EHP) and National Capital District (NCD).

A semi-structured interview guide was developed (Additional file 1) by the lead author (EG), an East Sepik woman who has worked in the health sector and lived in NCD for over ten years. The guide was field-tested by EG in November 2013 with one focus group, and then adjusted with input from co-author (RG) prior to subsequent use. The interview guide was developed in English, translated to Tok Pisin (a lingua franca of PNG) and back-translated to ensure accuracy and consistency [30]. Data were collected by EG who used existing relationships with World Vision PNG, NCD Youth Office, Anglicare PNG and personal networks to identify and recruit participants. Participants who were the service recipients from these three organizations were identified and recruited by research assistants using the recruitment criteria. All focus group discussions and interviews were recorded using MP3 audio recorder and transcribed later. Interviews lasted between 45 and 65 min each. EG was supported by two PNG research assistants who took field notes and transcribed focus group discussions (FGDs) and individual interviews (IIs).

**Fig. 2:**
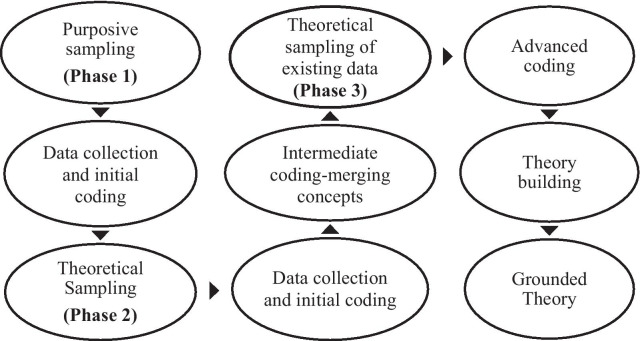
Process of sampling, data collection and analysis to construct grounded theory

FGDs and IIs were facilitated to explore women’s pre-menarcheal understandings of the meaning of body changes and menarche, social and cultural beliefs, perceptions and practices, and how girls are prepared for womanhood. The languages of Tok Pisin and/or English were used in FGDs and IIs, depending upon preference of participants. Purposive sampling of participants (Phase One) (Fig. 2) was followed by theoretical sampling (Phase Two). Women who were born, raised and had menarche in the four study sites were eligible for inclusion. The nominated age range for participants was 13–44 years. This age range includes post-menarche young and older women with personal experiences of menarche who are able to share stories. Cultural diversity, varied cultural beliefs and practices about menarche, sexuality, menstruation, and the evolving modern lifestyles were considered. A total of 98 women participated in FGDs (n = 10) and IIs (n = 6) during 2013 and 2014. Homogeneity in socio-cultural and educational status and age was sought for FGDs so that participants felt comfortable to share their stories [31]. FGDs were facilitated with young women (13–25 years) separate to older women (26–44 years). Women of 45 years and over also volunteered to attend—these women could not be refused participation because it was culturally inappropriate for EG, as a PNG woman, to do so. Elizabeth Gumbaketi (EG), lead researcher is a senior PNG indigenous woman with many years of experience in Public Health in PNG, and who experienced menarche in a village setting in PNG. The remaining authors have demonstrated an ongoing commitment to the improved sexual and reproductive health and wellbeing of women and girls in Pacific island nations, and other settings.

The demographic characteristics of participants is shown in Table 1. Due to resource constraints for a PhD study, all data were collected and later analyzed following the process described in Fig. 1.

**Table 1:** Socio-demographic and study characteristics of participants

Study sites (provinces)	Data collection strategies	Interview number	Total participants number	Participants’ distribution by age range		
				13–25 years	26–44 years	> 45 years
East Sepik	Focus Group Discussion	4	27	10	9	8
	Individual interview	3	4	2	2	0
Eastern Highlands	Focus Group Discussion	1	8	3	3	2
	Individual interview	0	0	0	0	0
Milne Bay	Focus Group Discussion	2	25	11	10	4
	Individual interview	3	4	0	1	3
National Capital District	Focus Group Discussion	3	30	12	17	1
	Individual interview	0	0	0	0	0
Total		16	98	38	42	18

Data collected from FGDs and IIs were coded after all data collection events by EG, using N-Vivo Plus software (Version 11). Initial coding was conducted following data collection due to constraints in the field environment. Intermediate coding was then conducted to merge key concepts identified in initial coding. Theoretical sampling (Phase 3) was used to further interrogate existing data. EG, with support from MRM, elevated existing codes to focused codes that became more refined and abstract. Iterative and constant comparative methods and continuous memoing were used during analysis to identify relations, linkages and enable the elevation of key concepts to build a theory from the data [30, 33, 35]. The process continued until the core category, four interconnecting sub-categories and the intervening conditions were identified. To increase theoretical sensitivity, and as part of the scholarly requirement for a PhD, a literature review was in part conducted prior to the study and then completed after data collection and analysis. To ensure credibility and accuracy of the grounded theory, the findings were presented at two conferences where PNG women were present and invited to provide feedback. PNG women conference attendees were asked if the constructivist grounded theory model comprising the core category of ‘Making of a Strong Women’ and the inter-connecting sub-categories appropriately explained the experiences of young PNG women at menarche. The responses from women attending demonstrated ‘grab & fit’. The concepts of ‘grab’ and ‘fit’ assess the quality of the grounded theory—‘grab’ enables the reader or listener to understand the idea and what is going on while ‘fit’ ensures meaningful links between concepts and the data [64].

### Ethical considerations

Ethical approval for this study was obtained from the Papua New Guinea Medical Research and Advisory Council (reference number: MRAC13.40) of National Department of Health, Papua New Guinea and Human Research Ethics Committee (reference number: H5317) of James Cook University, Australia. All participants gave consent before participating in the study. Written consent was obtained from those able to read and write. Some women who were illiterate gave their fingerprints as a consent for study (as approved by ethics committee). Parental consent was obtained for participants under the age of 18 years as per the approved ethics process. Written information sheet and consent forms were sent to each participant before the study to read, understand and make an informed decision to participate in the study. For illiterate women, the study was explained before the interview to enable informed decision before participation in the study.

## Results

The constructivist grounded theory of ‘Making of a Strong Woman’ explains the rationale for the social and cultural process that happens to young naïve girls at pre-menarche and menarcheal stage in order to ‘Achieve Womanhood’. Young naïve girls are those who have no previous personal experience of menarche or menstruation. The key findings in this study suggest that the experiences of girls are rooted in social and cultural beliefs and practices around menstruation and patriarchal perceptions of menstrual blood as polluting. Although the experiences are traumatic, the isolation and initiation process provides a valuable traditional educational asset for preparing young naïve girls for womanhood in the society that restricts open communication about private body parts such as ‘vagina’ or ‘breast’.

**Fig. 3:**
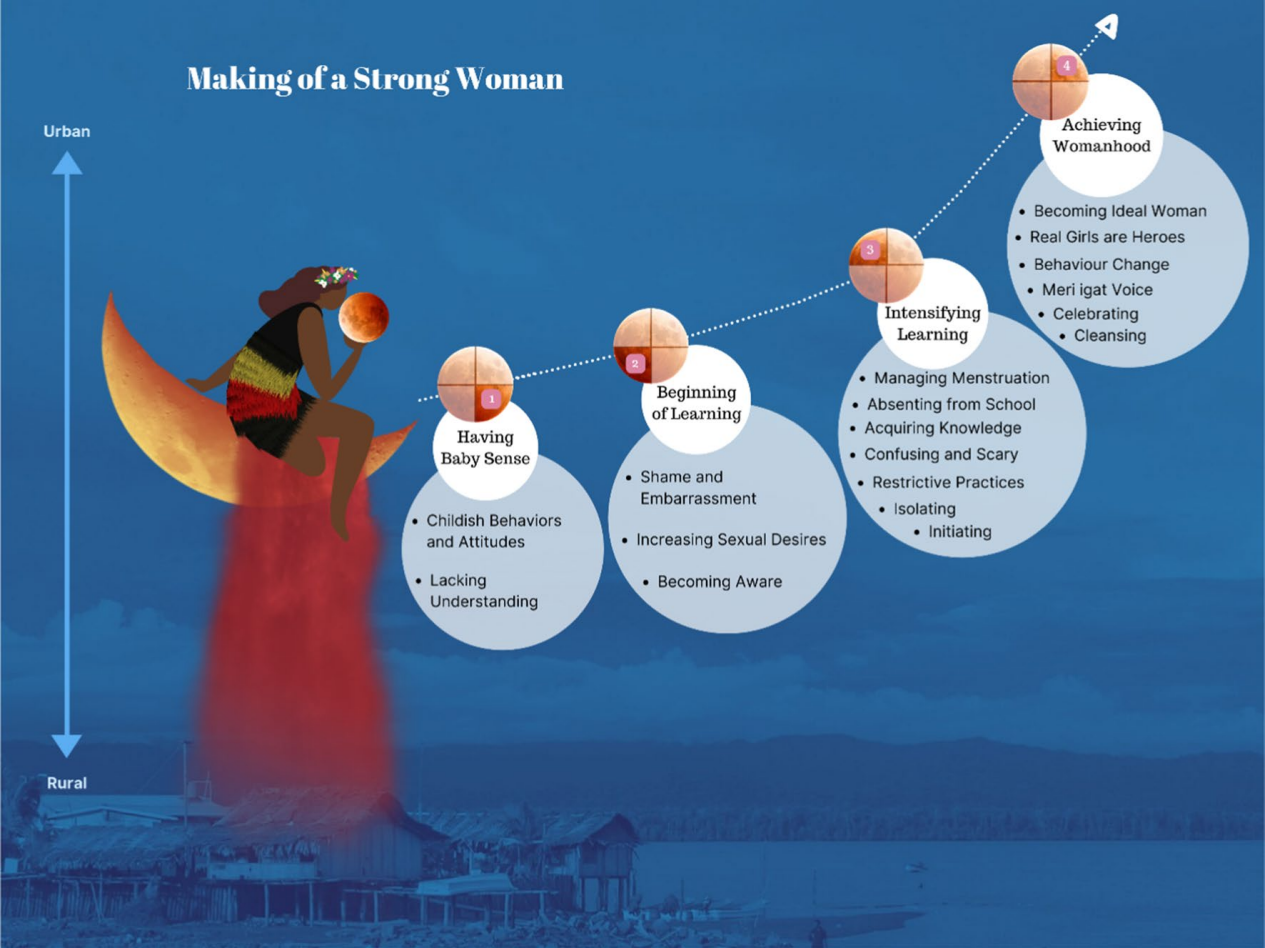
Theoretical model of ‘Making of a Strong Woman’

The core category of ‘Making of a Strong Woman’ with four interconnected categories: ‘Having Baby Sense’; ‘Beginning of Learning’; ‘Intensifying Learning’ and ‘Achieving Womanhood’ emerged through the inductive analysis. The four categories with the properties and intervening condition are explained in sequence according to the theoretical model (Fig. 3).

### Making of a Strong Woman

‘Making of a Strong Woman’, the core and overarching category represents the phenomena that connect all concepts, categories, properties and their intervening conditions and explains the experiences of having *fes sikmun* (menarche, which is literally ‘first sick moon’) in Tok Pisin.

The core category of ‘Making of a Strong Woman’ explains why and how the young naïve girls undergo the social and cultural process at menarche. Women explained that the rationale for ‘Making of a Strong Woman’ is to create awareness about their body changes and to acquire the status of womanhood in a complex physical and cultural environment they live in and where open communication about sexuality is shameful. “…*Em tambu ya…em samting blo sem,…em castom blo mipla* (it’s not allowed…it’s a shameful thing… it’s our customs) [young woman, FGD-02, NCD]. Given this systematic lack of open communication, the society uses isolation, initiation and ritualized cleansing processes associated with cultural beliefs as a way of preparing girls for womanhood.

These processes are traditionally established, passed down through generations and are still active in many cultures [8]. The young naïve girls are put through a culturally rigorous process to cause them to become aware of their changing body, menarche and the womanhood obligation. “*…long strongim mipla, ol bai putim mipla long haus na toktok lo mipla….Na mipla bai kisim strong…olsem kamap mipla meri… na mipla bai kamap strong* (To make us strong, they will put us in the house and talk to us…. Then we will acquire the strength…and become aware of our womanhood… and we will become strong)” [Older woman, FGD-01, ESP]. Though isolation, initiation and cleansing processes can be associated with harmful practices, these practices are understood to provide a necessary learning opportunity for creating awareness for menarcheal girls about their body changes, menarche and the obligations for womanhood in cultures in which open discussion about sexual topics are restricted.

The process for ‘Making of a Strong Woman’ is entrenched in the cultural beliefs and practices around menstrual blood and its implications for men and food gardens, and cultural expectations of an ideal woman. Menstrual blood is perceived as harmful and bad, “*…sikmun em nogut…em posin…bai bagarapim ol man* (menstrual blood is bad…it’s poison…it will destroy men)” [Young woman, FGD-02, ESP]. Thus the menstruating young women must be segregated to avoid contamination of male figures including food gardens and communal places. The restrictive practices are associated with the cultural perception of menstruation blood as polluting.

‘Making of a Strong Woman’ has two key concepts; “making” and “strong”. Women referred to “making” as a process of creating (*wokim*) and “strong” as having power to move heavy weights (*igat strong long karim heavy*) or perform other physically and emotionally demanding tasks (*wokim strongpla wok*). “*Taim ol wokim custom lo mipla em olsem…bai body b'long mipla kamap strong long wokim strongpla wok olsem garden* (When they do custom to us, it’s like …our bodies will be strong enough to do tough jobs as gardening)” [Elderly woman, FGD-02, ESP]. The concepts of “making” and “strong” underpin the social and cultural processes practices for creating an ideal woman in PNG. An ideal woman is expected to possess qualities that are valued in woman by society.

‘Making of a Strong Woman’ is a collective process that involves extended family and community members. Isolation, initiation and cleansing ritualized practices provide an opportunity for focused teaching and preparation where immediate and extended female family members are involved. Men and women involved in the isolation and initiation process have distinct cultural roles and responsibilities. Grandmothers, aunties and in some situations, mothers, are typically involved in the teaching of and caring for menstruating girls, while fathers, uncles and grandfathers are responsible for decision making, building menstrual huts, toilets and initiation rituals.

### Having Baby Sense

‘Having Baby Sense’, the first category of the grounded theory for ‘Making of a Strong Woman’, refers to the childhood stage. This stage is characterized with innocence, childish thoughts, behaviors and attitudes, childish activities, lack of abstract thinking and concrete ideas and observations.

Most women reported that, during childhood, they lacked knowledge and understanding about the meaning of body changes and menarche and the expectations that are associated with these changes. The girl child’s lack of understanding was explained as them being too young to understand and make sense of what they saw, observed and heard. Furthermore, the women explained that, as children, they could not understand and grasp concrete ideas and knowledge because they had “*bebi sense* (baby sense)” which is an in-vivo code from this statement *“Mi bebi sens yet… nana blo mama yet na mi no save long wanpla samting yah* (I had baby’s sense…my mother’s little baby and I did not understand anything) [Young woman from settlement, FGD-02, NCD]. Similar statements were echoed by women throughout the study when discussing their childhood stage. An educated woman from one focus group discussion said, *“We could not really connect the information. Information was not clear”* [FGD-01, NCD]*.* Consequently, the girls were unable to understand and make sense of what was happening to their bodies. Some women recalled hearing and observing their mothers having menstruation, but did not understand. Some expressed feeling lost or confused. “*Ol tok sikmun na kain olsem, mi no bin save.* (They talked about menstruation and all that, but I had no idea)” [Young educated woman, FGD-03, ESP].

Lack of understanding amongst girls is linked to cultural secrecy, shame and taboo. Most women explained that open discussion about *sikmun* (menstruation) and private body parts is “*tambu”* (taboo) and a shameful thing to discuss with children. *“…we are not allowed to talk to our daughters about private body parts…it’s a shameful thing…it’s our culture)* [young educated woman, FGD-02, NCD]. Consequently, most women explained that mothers were unable to inform them about body changes and menarche while they were growing up.

‘Having Baby Sense’ is also characterized with childhood behaviours and attitudes that are expected to cease before the girl achieves womanhood. *“…we play a lot…when we are still baby…when we grow, our parents tell us to stop acting baby baby”* [Older woman, FGD-02, MBP). There is an expectation that a girl child is expected to change their childhood behavior and attitude when physical bodily changes happen. While growing up, girls are expected to help with domestic duties such as babysitting, collecting firewood, fetching water for family meals and doing dishes. These practices are aimed at instilling in the female children the expected duties of womanhood.

‘Having Baby Sense’ is conceptualized as an important cultural premise for ‘Making of a Strong Woman’ because of the girl child’s lack of abstract and logical thinking at childhood, demonstrated in their inability to understand, comprehend and make sense of their body changes. Furthermore, cultural taboo, shame and secrecy about open communication on sexuality prevents girls from learning about their body changes in childhood. Moreover, traits typical of young girls such as 'not knowing' are culturally undesirable traits in a mature woman. The girls are expected to develop into mature and strong women who can perform social and cultural obligations. Therefore, cultural systems and processes are put in place to eliminate those childhood characteristics from 'having baby sense' and to prepare girls with the knowledge and skills needed to become strong women.

### Beginning of Learning

‘Beginning of Learning’ is the second category of the constructivist grounded theory. Breast development is a physical marker that represents a stage of puberty and reproductive maturation. A girl’s experience of breast development is characterized by lack of knowledge, feeling scared and embarrassed, indirect learning, family mobilizing support, increased sexual feeling and teasing and ridiculing. In PNG, breast development is a social marker for maturation that causes families to increase protection of young girls and commence marriage preparation. *“Em wei blo mipla…taim susu kamap nau, ol mama papa i tok…yu kamap meri nau* (that’s our way…when breast starts developing, parents say…you are becoming a woman now)*”* [Young woman, FGD-02, EHP]. Women also mentioned that breast development stage is when families start preparing for initiation practices at menarche.

Women lacked knowledge about the significance of their body changes, including menarche, at breast development. As explained under the previous category (‘Having Baby Sense’), lack of knowledge is also linked to cultural secrecy, shame and taboo that prevents open communication and support from many mothers. The culture of shame and secrecy was common in uneducated mothers from both urban and rural areas. However, education helps to increase a girl’s awareness of her changing body, including menarche. Parents with a formal education were better positioned to advise young girls about the meaning of breast development and pre-menarche preparation, when compared to uneducated parents from both rural and urban areas. Women who received advice from their parents and family members, mostly had parents and family members who were educated, living in urban areas had some understanding about the biology of reproductive organs. Parents with formal education were in a better position compared to uneducated parents from both rural and urban areas, when advising the young girls about the meaning of breast development and pre-menarche preparation. A young woman from East Sepik Province said, *“…Mi, olsem* (Like, for me)*, I was lucky because my sister who was a school teacher told me”* [Young woman, FGD-01, ESP]. Women also explained that some uneducated women from the village are unaware of the functions of the reproductive organs and therefore feel uncomfortable to engage in discussions about body changes and menarche with their daughters.

Women who attended schools expressed that formal education did not always result in girls knowing about body changes. This lack of knowledge contributed to a girls’ lack of understanding of and unpreparedness for menarche. One young woman explained that, even if sexual and reproductive development was taught in schools, for many girls, this occurred after menarche. *“I was taught about anatomy and physiology… in Home Economics class but it was too late…I had menses already”* [Young working woman, FGD-02, NCD]. A woman from a focus group discussion in MBP heard about menarche and how to manage menstruation from the dormitory meetings in her high school boarding house, yet lacked confidence about how to manage menstruation.

Breast development is a stage when girls may undergo a period of emotional and psychological upheaval. Many women recalled feeling embarrassed, strange and scared, and withdrew themselves from family, friends and social activities when they became aware of “*susu sanap”* (breast development). *“I was surprised and felt ashamed because I did not understand”* [Young woman, individual interview (II)-02, ESP]. Women felt embarrassed due to teasing, staring and gossiping, commonly from males, friends, family members and other women. Although women from all study sites experienced shame and embarrassment, the women from NCD were worst affected by teasing, staring and stalking behaviors of others. Women also recalled that at this time, they started developing feelings and attraction towards males. Many women explained that they were scared and embarrassed about their breast development because they were unaware of when it would occur.”*…Mi ino save tu (I did not know). When my breast developed I was shamed”* [Young woman, FGD-01, ESP]. Women also spoke about how being stalked by boys, especially after school, negatively affect their school attendances.

The breast development stage was characterized by indirect forms of communication and learning for most women in the study. Women began learning through indirect forms of communication due to cultural secrecy, including shame. Indirect forms of communication and learning happened through observation, stories and gossip, media, jokes and teasing, harsh words, scolding, myths, parables, metaphors and analogies, including extreme means of communication such as death threats. Women reported these modes of indirect communication and learning, although common, varied between each of the four provinces. Metaphors were commonly used in Eastern Highlands, and East Sepik Provinces; death threats were more commonly reported in NCD. Death threats were made to inform the girls that did not easily adhere to advice about the potential risks associated with sex and unplanned pregnancies that could potentially bring shame and embarrassment to the family. In Milne Bay Province, women learnt about their body changes through jokes by elderly women without formal education. Recalling her experience, a middle-aged woman from Milne Bay Province explained that, *“As for me, God was good to me. He gave me wisdom. There was no education. So I was learning through the jokes”* [FGD-02, MBP]. This method of communication and learning is unclear and often leads to confusion and shame.

Metaphors, slangs and analogies were commonly used in an effort to explain and inform the girls about body changes, including menarche. However, women explained that the use of metaphors were usually vague, and too unclear to adequately understand. An example of metaphors from Eastern Highlands Province is: *“Kiau blo yu bai buruk* (Your egg will break)” [elderly woman, FGGD-01, EHP]. Metaphors and analogies are used to avoid direct communication about private body parts due to the culture of secrecy and shame. Some women also recalled learning from scolding and swear words such as ‘cunt’ or by teasingly describing breast changes by family members in an effort to make them realize that their bodies are changing.

Women learnt about their changing bodies through being teased and ridiculed, and commonly reported feelings of shame and embarrassment. Consequently, girls avoided participating in social activities, including school attendance, for fear of being teased and ridiculed. Most women recalled being teased after their breast buds started developing. Distressing and demeaning comments such as “tomato meat” were used to describe the young girls’ developing breasts. Despite their distress, the girls developed some understanding about the sexual meaning of their body changes. A young women from ESP said, *“Ol boys, taim ol tisim you, ol bai kisim yu stret lo bun. (When boys tease you, they’ll get you good and proper)”* [FGD-04, ESP]. This quote implies that teasing from boys often leaves the girls feeling very embarrassed but also more aware of bodily changes.

Sexual feelings by girls towards the opposite sex also increased at breast development and before menarche. Women from all provinces (ESP, EHP, MBP, and NCD) spoke a lot about the importance of family support towards them after their breast buds started developing. Women expressed that family support is increased to prevent girls from early or pre-marital exposure to sexual activity. However, communication taboo (discussed earlier) about sexuality prevents direct transfer of information about meaning of changing body including associated risks leaving many young girls vulnerable to sexual reproductive risks and consequences. Women from MBP spoke specifically about a cultural practice in Trobriand Island culture where girls are exposed to traditionally accepted practice of pre-menarche sexual initiation. This practice sometimes lead to stigma at the onset of menarche because of the false perception that a young woman’s menarcheal state is due to early sexual intercourse related to that cultural practice. Women in NCD compared the sexual risks associated with breast development and expressed that young girls in their district are becoming vulnerable to early sexual exposure due to economic pressures. *“Ol meri blo nau ya,…taim susu kamap tasol…ol sanap pinis lo rot …long kisim mani (Girls these days…when breasts starts developing…they are already on the road…to get money)”* [Older woman, FGD-03, NCD]. This quote refers to transactional sex: exchange sex for money or goods in order to survive.

Death threats, although uncommon, were reported by a few women from NCD. These threats are used in an attempt to prevent girls especially from risky sexual behaviors associated with selling or exchanging sex and teenage pregnancies. *“…If you make any silly mistakes and you become pregnant…they threaten us. They say, you don’t stay in this house, we’ll kill you”* [Older woman, FGD-03, NCD]. Although death threats are considered harsh, the girls are forced to learn to protect their bodies from pre-marital sexual exposure and teenage pregnancies.

Preparation for menarche by members of the girl’s family commences at breast development in both urban and rural areas. Preparation usually includes making gardens, preparing food and finding the money to host the cultural practices, feasts and celebrations associated with *fes sikmun* (menarche). During this time, the girls are sometimes informed about body changes and menarche indirectly by family members. However, most times, women start to realize the significance of their changing body when their family members use their changing body as a cue to commence preparation of girls for menarche.

### Intensifying learning

Intensifying learning is the third category of ‘Making of a Strong Woman’. Intensifying learning is characterized by onset of *fes sikmun* (menarche), isolation and initiation rituals, focused and intensive learning. It was conceptualized from the study that, the three important processes of isolation (first step), initiation (second step) and cleansing (third step) were the essential traditionally-instituted learning processes for preparing young naïve girls for womanhood. Although these three cultural conceptual processes across all the study sites, the extent of their application greatly varied in the accounts of women from urban (NCD) and rural study locations (ESP, EHP and MBP). In Milne Bay Province, most women explained that they did not go through the full process of isolation, initiation and cleansing due to religious influences or that their culture did not have such practices.

**Fig. 4:**
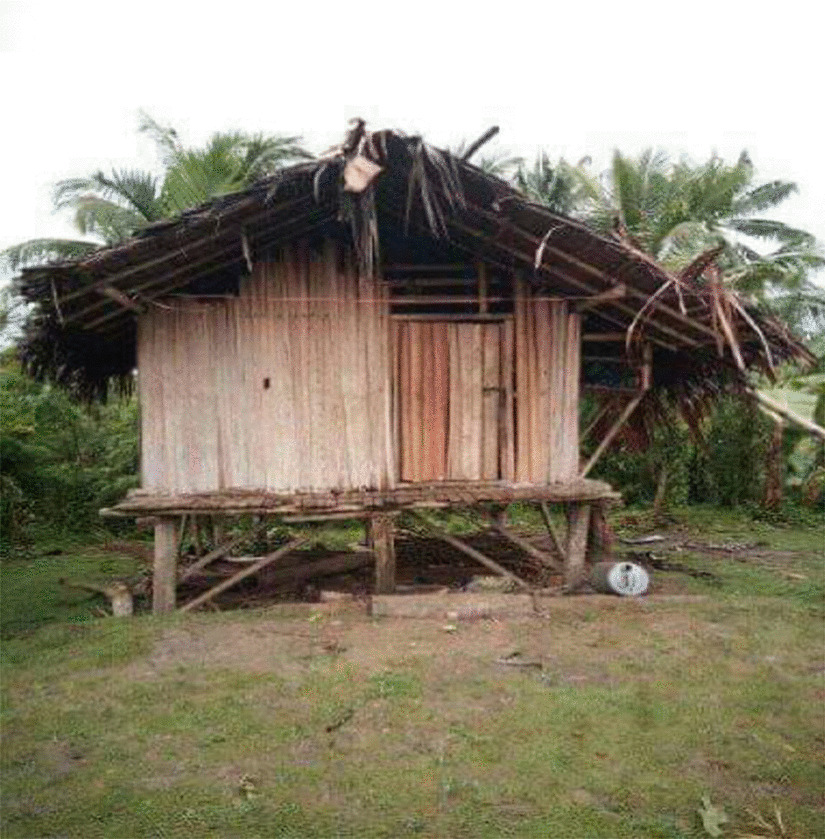
*Haus-meri* (menstrual hut) in Maprik district, East Sepik Province, PNG

Isolation happens immediately at the onset of menarche. In rural areas, isolation takes place in *haus-meri* (women’s only house) typically built for menstruating women a few meters away from the family house. Because of the cultural significance of this house, different cultures have different names. Women from Maprik in East Sepik Province call this house *wa’nga nga, or simbai* [FGD-01, ESP]. Figure 4 shows a typical *haus-meri* in Maprik District, East Sepik Province. This is a special house accessed by older women, however women reported that young pre-menarcheal prepubescent girls are also allowed. In other locations such as Russel Island in Milne Bay Province, the women explained that menstruating girls and women are sent to live in the bush until the menstrual flow ceases. A working woman from NCD reported that due to lack of family support and facilities, she was isolated in a bedroom inside the family home experiencing a limited application of cultural beliefs and practices. *“…I was isolated in the girl’s bedroom…not like in full…like in village”* [FGD-02, NCD].

Immediate isolation is linked to cultural perceptions of menstrual blood as harmful, potentially bringing bad luck to men and destroying food gardens. Women were immediately isolated at menarche to avoid contamination, *“Blut ya…em nogut (menstrual blood is bad)”* and can “*daunim strong blong man* (reduce men’s strength)” [Young women from NCD, FGD-02]. The practice of immediate isolation was traumatic for most women in the study. Many women spoke about feeling scared and embarrassed at the onset of *fes sikmun (menarche)* because they were unprepared. Many women felt scared and confused when they were immediately taken into isolation. *“First taim blo mi too na mi poret na faul ya,* (It was my first time. That’s why I was scared and confused)” [Young woman ESP, II -03]. Several girls spoke about being prepared from observing the practice from their older siblings and other women, however, they still felt scared when they experienced menarche.

Women conceal their menstruation status due to shame from myths and rumors. Women in Milne Bay Province explained that young girls in Trobriand Islands culture (MBP) conceal their menstruation status at menarche from mothers due to myths and rumors that, the onset of menarche is a consequence of early sexual exposure during a cultural festival called ‘*Mila Mila’*, and that this outcome would mean they would be scolded and stigmatized. The women explained that sex is ‘free’ during the ‘*Mila Mila*’ festival where pubescent and pre-menarcheal girls are culturally encouraged to be participate as a socializing event to celebrate the yam harvest. *“…They wait for that event. They are free to just go and do anything. Do all the sex they need…before they are matured… say 10, 9 years old, they are already into this Mila Mala thing (referring to festival)*” [Older woman, MBP II-03]. Shame also causes young girls to conceal their menstrual status. Many women recalled their experiences and said, they did not tell their parents especially mothers when they had menarche because of shame.

Women spoke about various practices that happened in isolation, including cultural initiations and rituals. Types and scope of those initiation practices varied between the four study provinces. Cultural practices were common in the stories of women from Eastern Highlands and East Sepik Provinces compared to Milne Bay Province. Although women from NCD reported less involvement in cultural practices, two women reported undergoing intensive initiation and cleansing rituals. These two women described how and why the cultural practices happened. One woman from ESP who had her *fes sikmun* in an urban area said she underwent whipping and scarification initiation which was physically and psychologically traumatizing. However, her father believed that the practice was necessary to make her realize her body had changed into a woman and she should behave like a woman and not like baby anymore. Despite her initial resistance, the young woman later appreciated the cultural practice, because she understood that the initiation practice caused her to appreciate her new status and expectation that went with body changes and menarche; *“Em castom ya. Ol mekim disla ol samting lo mi na mi kamap meri. Strongpla meri (It’s our custom, I underwent the initiation ritual, and that made me a strong woman*)” [FGD-03, ESP].

Preparing young girls for womanhood is a communal activity in the family where different members of immediate and extended families are involved. The grandmothers and aunties play significant role by supporting mothers to have direct communication with the menstruating young woman about the meaning of body changes, *fes sikmun*, sex and reproduction, childbearing, menstruation management and changing roles and responsibilities and expectations of womanhood. This conversation is commonly done in seclusion in the menstrual hut. Women also spoke about learning skills of making *bilums* (carry bag). *Bilums* are traditional bags used for domestic purposes such as transporting food from food garden and rocking babies to sleep. Grandmothers and aunties are post-menarcheal women, who had previously gone through isolation and initiation practice, are respected by the family and community, and have cultural obligations to prepare the girls. While mothers are able to provide support, their involvement is often limited due to the shame. It has been conceptualized that, because of cultural shame, the responsibility of mothers in the preparation of their daughters for womanhood becomes a communal activity. Women explained, the aunties and grandmothers are able to talk to the menstruating girl because it was their cultural and family obligation. *“Mi salim pikinini meri go lo tumbuna… lo ples… ol bai toktok lo em (I sent my daughter to grandmother… to the village…they will talk to her)”* [FGD-02, NCD].

Certain restrictive practices were enforced on the menstruating young woman. During isolation and initiation process, food taboos, cooking restrictions, bathing restrictions and social isolation are enforced. Young menstruating women were not allowed outside of the house and could stay in isolation for as long as is decided by the father of the young woman. In terms of managing menstruation and keeping clean, women expressed that bathing restrictions prevented them from cleaning blood flow while in isolation. However, there were mixed perceptions and beliefs about bathing restrictions in urban areas. While some adhered to the bathing restrictions, others did not due to increasing level of awareness about the negative implications of poor personal hygiene on girl’s health. One educated women from NCD said, *“…I was allowed to clean myself but not to expose myself but to only stay in my room.”* [FGD-02, NCD]. The women who went to high school spoke about not having adequate water for cleaning themselves and difficulties of disposing soiled pads. Women spoke about feeling unclean and having a strong smell from blood but despite probing, they did not report (and maybe were not aware of or culturally unable to) developing infections such as rashes. Women appeared to feel shy and chose not to talk when direct questions were asked about private body parts.

Different methods are used to manage menstruation. Old cloths, towels, menstrual pads, traditional materials such as moss and leaves are utilized. In urban areas, women spoke about using modern commercially available menstrual pads but these are sometimes inaccessible due to cost and the shame of purchasing them in public. Some of these barriers are also experienced in the rural areas. Modern menstruation management materials such as sanitary pads are usually unavailable in rural areas. This may be due to a lack of supplies in local stores or that women lack access to money to buy the materials. Women further explained that, the menstrual pads are seldom available due to shame by the retailers or shop owners to sell these products. Women in some rural areas still use traditional materials to manage menstruation. Women from the EHP described making a soft traditional pad out of moss to absorb blood, and this method is still being used by some women in villages who do not have access to modern materials. Some women said, they did not use anything, which was always leaking and uncomfortable and can be embarrassing.

Disposing soiled menstrual management product is difficult because of the unavailability of sanitary facilities in both rural and urban areas. In urban areas, many women expressed that, they did not have the facility to dispose soiled sanitary pads. Putting soiled items in rubbish bin was the only option but are sometimes dug up by animals such as dogs. *“…ol dog save digim na rausim…sem ya* (…the dogs dig and remove them…it shameful)” [FGD-01, ESP]. Few women who boarded in high school burnt soiled pads at night when people were not watching because of unavailability of disposal facilities. In rural areas, women explained different ways of managing soiled materials. Private toilets are built for menstruating women. Others keep their soiled materials during the isolation period and are burnt after isolation process.

The isolation and initiation processes, as explained under this category, provides intensive and focused learning for young woman. After the cultural process of isolation and initiation, the young woman goes through a ritualized cleansing process to achieve womanhood.

### Achieving womanhood

‘Achieving womanhood’ refers to the cleansing practice. Cleansing practice is the third phase of the cultural practice after isolation and initiation practice. At the cleansing phase, the menstruating girl undergoes a ritualized cleansing process to rid them from the status of being dirty and contaminated, in order to achieve womanhood status. The cleansing process signifies that, the young woman is now free from menstrual pollution. Women also explained that, after the cleansing ritual, they became fully aware of the meaning of their body changes and menarche, including the social and cultural expectation that is associated with the change in status from girlhood to womanhood. *“Em olsem custom em bikpla samting ya. Ol wokim disla na olsem yumi kamap olsem meri (The custom is a big thing. They did the custom and we able to become a woman)”* [Young woman, FGD-03, ESP]. Women who went through the cultural practices of isolation and initiation explained that they felt psychologically prepared to assume woman’s role including marriage and childbirth.

At ‘Achieving Womanhood’, the young women are expected to change their attitudes and behaviors and start performing new roles and responsibilities including domestic chores such as cooking, cleaning, fetching water, and gathering wood. The woman is now expected to be able to manage gardens independently from her parents. These are the roles and responsibilities, and cultural expectations in marriage, that are explained to the woman by the grandmothers and aunties while in isolation. ‘Achieving womanhood‘ is usually marked with celebration that include feasting, singing, dancing, rituals and chanting, rewarding, giving gifts, traditional dressings and beautification. Celebration is a show of a family’s happiness that their daughter has reached maturation and achieved womanhood. *“Ol sa hamamas lo pikinini meri grow up na kamap meri nau* (Celebration is a show of happiness that their daughter has become a woman)” [Individual Interview-03, ESP].

Cleansing practices vary according to different cultures within PNG. While water is commonly used to signify cleansing, the rituals are different according to different cultural context. For example, women from ESP spoke about being carried out of the house and into the river and washed. *“Ol wasim nau, em olsem mi klinim… Ol i bilip olsem taim mi kisim sikmun em mi kamap olsem dirty* (They washed me, then it is like, I’m clean. They believe that, when I have my menstruation, I became dirty…)” [ESP, FGD-01]. In the Eastern Highlands Province, girls jump over a deadwood and break sugar cane before being washed with warm water. After cleansing, the young woman is provided gifts followed by traditional dressing, singing, dancing, feasting which usually include extended family members. In NCD, women spoke about washing with water which was followed with celebration and gifts from families. In Milne Bay Province, women spoke about being cleansed using sea water followed by giving of gifts. *“She went straight into the sea, washed, came out and rinsed her body with warm water. And then they gave her buggies and dressed her up with buggies*^1^* and made a big feast”* [MBPFGD02].

Marriage is considered the ultimate reason for girl’s initiation and preparation for womanhood. This perception was shared by most women from all four provinces. One women from Milne Bay Province expressed that *“real girls are heroes”* [Individual Interview-02, MBP] to describe the cultural perception of an ideal strong woman. The expression asserts competition between young women about who can remain unmarried and abstain from pre-marital sex until marriage. *“Sapos em stap longpla taim em trupla meri* (if she stays for long period of time, she’s a real woman)*”* [MBP, Individual Interview-02]. Unmarried women without pre-marital sexual exposure are valued. Sex is only considered appropriate in marriage and sex outside of marriage is considered culturally inappropriate. Girls who engage in pre-marital sex outside of marriage and end up with teenage pregnancy outside of marriage is a taboo that can bring shame and disrepute the family. The girl can be stigmatized for unplanned teenage pregnancy outside of marriage. *“Igat bikpla tambu long karim bel nating (There’s a big taboo with pregnancy outside of marriage)”* [ESP, FGD-04]. However, women also explained that, the perception about marriage being an ultimate rationale for initiation practice is changing with improved education and changing lifestyle. Not all women going through female initiation are prepared for marriage. Some parents allow their daughters to go through the female initiation process not only for marriage but to instill good and respectable characters and behaviours of being an ideal woman. *“…Ol wokim olsem bai mipla respectim ol bikpla ol lain, harem tok blo mama papa. Yu (mipla) mas noken sakim tok* (they do this to us so that we can learn to respect the elders and parents. Not to disobey them)” [young woman from ESP, FGD-03].

Characteristics of strong women are culturally defined. The common characteristics of a strong or good woman include a fat and shiny body, non-participant in pre-marital sex, physical fitness and respect for elders and men. *“Mipla mas respectim ol bikpla ol lain, haren toktok na halivim papamama…bodi blo mipla mas kamap shine* (We must respect the elders, be obedient and help our parents…our bodies must shine)” [FGD-03, ESP]. In Eastern Highlands Province, girls are told to change their attitude and behaviors, and are encouraged to gain weight while they are in isolation to reach the desired body form before “graduating” as a woman.

Women also spoke about the risks of not being effectively prepared to become a woman. Some women explained that girls who grow up in urban centers, without traditional support systems for preparing girls for womanhood will likely be vulnerable to risky sexual behaviors. One woman from NCD explained that, because the urban environment was not conducive to preparing her daughter for womanhood, she sent her daughter back to the village in the province to live with her maternal grandmother and uncle so that they will prepare her for menarche. Several young women from settlements in NCD reported they were unprepared at menarche because their parents were disconnected from their traditional cultures from being away from their villages for many years. A girl from Eastern Highlands Province living in Port Moresby, was saddened over the death of her mother because she had not been around to guide and support her at menarche. This young woman could not access her closest aunties and grandparents who were in the village for support and advice when she had menarche.

Women from East Sepik and Eastern Highlands Provinces spoke about the significance of the cultural roles played by relatives to prepare girls for womanhood. The women explained that the older women (grandmothers, aunties and other older women) who are involved in providing support to the girls in isolation are rewarded with money and in kind for their involvement. Men are also rewarded for providing support, which although not directly involved in talking to the girls, they are involved in decision making and organizing rituals, initiations and feasts.

## Discussion

This Constructivist Grounded Theory study found the experiences of young girls at menarche are underpinned by social and cultural processes that enable the ‘Making of a Strong Woman’. Young women’s experiences at menarche in PNG are linked to social and cultural beliefs, perceptions and practices around menstrual blood, culture of secrecy and shame, and the value placed on girl children for marriage and childbirth.

The explanatory power of this grounded theory is enhanced by the use of the theoretical code [30], Social Determinants of Health (SDH) [37, 38]. A theoretical code helps to integrate the grounded theory into the literature [30, 63]. The theoretical code of SDH expands an understanding of processes described in the Making of a Strong Woman theory by examining determinants including social gradient, stress, early life, and social support impacts on health outcomes. This grounded theory explains that location (rural or urban) and social status of girls results in a different experience of menarche. Extending this theoretical code, the theories of sexual and reproductive health rights [40] and gender inequity [38, 46] have also been explored and applied. Culture, as a source of strength, is centralized within the Indigenous People’s Health Rights framework [39] and highlights the importance of traditional cultural processes consistent with the findings of this study.

This study found that, menarche represents transition from childhood to womanhood, and readiness for childbirth. Menarche, a key function of the female’s reproductive system, is fundamental to sexual reproductive health [40]. To attain menstrual health and hygiene, as is every girl’s right, access to appropriate pre-menarche information, menstruation management facilities and materials is required [6, 29, 40, 43]. This study found the many girls did not have access to these types of support. Most women lacked knowledge about their changing body at childhood until puberty. This finding is consistent with a number of other studies, including from LMICs. For example, a study by Manoshi and Shantri in Bengaluru urban district, India, found that adolescent girls without prior information about menstruation felt helpless and confused about menarche which impacted upon their ability to manage menstruation hygienically [44]. These experiences, underpinned by the scarcity, stigma and lack of affordability of safe menstruation management materials are also similar to findings reported from Nepal and Bangladesh [50, 51].

Cultural processes of “Making of a Strong Woman” were found to have both positive and negative impacts on a young woman’s physical, social, emotional and psychological wellbeing, including school attendance. The negative implications resulted from lack of pre-menarche awareness and preparation for menarche, social isolation and initiation practices, and restrictive beliefs and practices imposed on young girls because of her menstruation status. Feelings of shame and embassment at menarche have been reported in other studies conducted in PNG [5], Malawi in Africa [44], Bangladesh [50] and Nepal [46]. Cultural beliefs and initiation practices described in these studies are informed by a belief that menstrual blood is harmful [5, 27]. These beliefs are perpetuated by men who believe menstrual blood can reduce men’s strength, wellbeing and harm food gardens. These findings are consistent with undertaken in the PNG [5, 8, 10, 27] and other LMICs, documenting socio-cultural perceptions of menstrual blood and restrictive practices [2, 47–50].

The limited cultural practices at menarche in urban areas were linked to increased sexual risk-taking behaviours and consequences such as selling of sex and teenage pregnancies. These consequences were commonly reported by women from urban areas either as observations, or as women who had themselves experienced these consequences. This finding is similar to a study conducted in the Highlands of Papua, Indonesia [54], where unplanned pregnancy was linked to breakdown in traditional practice of preparing a girl child to manage her sexuality, pregnancy and childbirth.

Despite the sometimes negative experiences of girls at menarche, this study found that ‘Making of a Strong Woman’ is an important traditional social and cultural premise of preparing girls for womanhood. Characteristics of traditional cultural processes of isolation, initiation and cleansing for ‘Making of a Strong Woman’ resembles the process of rite of passage (rite of separation, rite of transition and rite of incorporation) initially described by Arnold van Gennep in 1960 [56]. The concept and the principle of the rite of passage are widely used in the development of various educational programs to facilitate adolescents’ transition to adulthood [57–59]. The conduct of such rites of passage for girls at menarche should be preserved, as they have been reported as beneficial for preparing girls for womanhood [68–72]. However, harmful elements should be replaced, upholding a girl’s human rights. This traditional learning model is an ideal practice to encourage social order, nurturing and instilling in girls an understanding of their body changes and menarche, menstrual health management and the expectations for womanhood. Western influences in PNG have seen the disintegration of such valuable practices [8, 27], which has been linked to increased rates of sexual and reproductive health issues among adolescents. In a study conducted by Jenkins in PNG, it was shown that initiation rites are important cultural institutions that provide a venue for adults’ to control the information flow to adolescents about sexuality, pregnancy and childbirth [27]. Initiation rites are used as traditional ways of learning in PNG. This approach to learning is based on traditional value systems of communalism and collectivism that is centered on reciprocal relationships—integral to Pacific cultures [66]. Westernized ways of learning are often formal, structured and hierarchical, for example as shown in Bloom’s taxonomy levels of learning [65]. Lack of attention to or maintenance of these important cultural determinants of health are contributing to the increasing SRH issues affecting the adolescents’ in Papua New Guinea today [27, 61].

### Implications for action

Findings from this constructivist grounded theory have implications for sexual and reproductive health education programs in the following areas to improve girls’ experience at menarche in PNG. The study found that, young girls lack awareness about the significance of their body changes and menarche, partly due to parent’s (especially mothers) inability to discuss sexuality education with their daughters due to shame and secrecy. Various educational opportunities such as early childhood learning programs or school health programs in PNG can be used to increase awareness of young pre-menarche girls about the significance of their body changes, menstrual health and hygiene and sexuality. Mothers should also be supported to have the ability to provide support to their daughters through various available educational opportunities including women’s networks or via the informal education sector.

Although fathers and other important male figures may not have direct role in providing awareness to the girls about body changes and menarche, they play a significant role in decision making. Fathers, men and boys should be provided education about the significance of body changes and menarche of females for them to understand and make relevant critical decisions regarding harmful cultural practices. Fathers, men and boys would then be able to support the young woman’s transit through menarche with dignity.

Cultural perceptions of menstrual blood as harmful is a discriminatory gendered belief that harms girls who are undergoing normal biological changes. The practice of isolation and initiation that stems from this cultural perception affects girls’ attendance at school, communal social activities, menstrual health management practices and nutrition. Despite the negative consequences of some of these cultural practices, the isolation and initiation process at menarche does provides an ideal space for intensified learning using menarche as the concrete subject for direct communication to girls. Women specifically suggested that the “*haus-meri*” concept be promoted and incorporated into positive intervention programs. The “*haus-meri*” concept requires further research as a strategic, health-promoting space for preparing young girls for womanhood in the contemporary PNG society.

### Limitations

This doctoral research was conducted with women from four different PNG provinces while they were living in Port Moresby in the National Capital District. This was due to the study being time bound and resource limited. This constraint was ameliorated by constant support from PhD advisors and in-country (PNG) research collaborators. Given the diversity of cultural and language groups in the 22 provinces of PNG, some of the study findings maybe not be relevant in different cultural contexts. Culturally-specific studies are required to understand the experiences of girls from different cultural contexts. Furthermore, this study did not included male’s perspective on girls’ experiences at menarche. Male perspectives are necessary to have an understanding of how males perceive menstruation in PNG and to determine action for positive change.

## Conclusion

The grounded theory of ‘Making of a Strong Woman’ explain processes as girls move from pre-menarche to womanhood in PNG. In PNG, many girls are unprepared for menarche. A lack of knowledge and understanding of body changes is due to cultural shame and secrecy surrounding open discussion about private body parts. Taboos around menstrual blood, which is considered harmful to men, causes stigma and discrimination. However, the traditional cultural processes of isolation and initiation do provide an opportunity to develop culturally appropriate intervention programs to address menstrual health needs of young girls in PNG. Cultural ways of knowing, being and doing are constantly adapting, and can be adapted in these circumstances to promote knowledge and understanding of menarche for young women in PNG.

The positive aspects of the traditional menarcheal rites can be used as a cultural strength to re-strategize the sexual reproductive health communication strategy. For example, mothers and the older female relatives of pre-menarcheal girls can be taught about menstrual health and hygiene to teach pre-menarche girls. Secondly, the positive elements of the three-stage process of isolation, initiation and cleansing can be used as a space to teach young girls about the meaning of changing body and menarche including other aspects of sexual reproductive health.

Discriminatory beliefs and practices should be addressed through improved and expanded sexual and reproductive health educational programs and policies. Because of the diversity in cultural and language groups in PNG, this study recommends that community leaders (including men), women and girls be involved in developing any intervention programs. Programs aimed at improving women and young girls’ sexual and reproductive health should include menstrual health and hygiene and take into consideration the social and cultural beliefs and practices around menarche.

## Supplementary Information

**Additional file 1.** Interview guide.

## Data Availability

All data used in this article are generated and analyzed from this study. All electronic data generated during this study is stored on a password protected drive at James Cook University, with hard copies of data stored at James Cook University, Cairns campus in a locked cupboard in accordance with the University’s data storage policies. Due to sensitivity of identifying the nature of the data, it is not in publicly available repositories but are available at James Cook University. Researchers wishing to gain access to the data may contact the corresponding author or College of Medicine and Dentistry, James Cook University, Australia.

## Abbreviations

MHH: Menstrual Health and Hygiene
LMIC: Low to Middle Income Countries
PNG: Papua New Guinea
EHP: Eastern Highlands Province
ESP: East Sepik Province
MBP: Milne Bay Province
NCD: National Capital District
SDH: Social Determinant of Health
FGD: Focus Group Discussion
II: Individual Interview

## Footnotes

1 Buggies are necklaces made out of shells and have monetary value. They are commonly used in Milne Bay Province.
