# Supplementary material for: ‘Making of a Strong Woman’: a constructivist grounded theory of the experiences of young women around menarche in Papua New Guinea
Source: BMC Womens Health. 2021 Apr 8;21:144. doi: 10.1186/s12905-021-01229-0 (PMC8034129; doi:10.1186/s12905-021-01229-0)
Supplement: Supplementary file 1 — Additional file 1. Interview guide. [file 12905_2021_1229_MOESM1_ESM.docx]

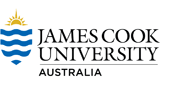


**INTERVIEW GUIDE**

**A. Young Women (Ages 13-25years)**

**INTRODUCTION:**

Hello, my name is (Elizabeth Gumbaketi) from James Cook University in Australia.

Thank you for taking time to participate in this Interview. This interview is part of the **group and individual interviews** that are being conducted in **(names of Province**) to learn about your experiences of having 1^st^ menstruation and what it means to **grow up and changing from being a girl to womanhood** in your local community in (**name of Province).**

Growing up into a young girl, having your 1^st^ period and experiencing bodily change are generally challenging for many young girls. Therefore, we want to understand how you personally felt when you had your first **menstruation.** This understanding will help us to work with respective government organisations to improve health services for young girls in Papua New Guinea.

You **(in FGD- are a group of young girls and women who)** have been purposefully selected in the village because you have the “lived” experience to be able to share your full experiences and stories of how it felt when you grew up from being a girl to being a woman in your local village or community in the **(name of Province).**

During this discussion, I **(name)** will ask questions and facilitate the conversation. Please keep in mind that there are no right or wrong answers to any questions that will be asked. The purpose is to stimulate conversation and to hear experiences of everyone in the room. There is no need to feel shame. I hope you will be comfortable to speak honestly, openly and share your experiences with us.

Please note that this session will be recorded **(and *(name)* will be taking notes)** to ensure we adequately capture your ideas and opinions during the conversation. Your name and comments from individual interviews can be kept confidential however, there is no guarantee that your name and comments from the focus group will remain confidential. Your name will not be attached to any comments you make. Furthermore, each of you are requested to respect each other’s opinion. Anything that is discussed in this room should remain confidential and not discussed outside of this building. Do you have any questions before we begin?

**(A): QUESTIONS**

1. Let’s do a quick round of introductions. Can each of you tell the group your name, where you come from, where you grew up?
2. **Tell me about the day when you had your first menstruation/period**.
3. **Probe**: What did you think?
4. **Probe**: Where were you when it happened?
5. **Probe**: That sounds interesting, please tell us more?
6. **Probe**: What happened?
7. **Probe**: How did you feel?
8. **Probe**: How did you manage menstrual flow?
9. **Probe:** Did it mean anything to you?
10. **Tell me about the day when you noticed physical changes in your body (Breast development, public hair, etc)?**
    1. **Probe:** What did you think?
    2. **Probe:** What happened?
    3. **Probe:** That sounds interesting, please tell me more?
    4. **Probe:** How did your parents, relative react?
11. **Who was the first person you told when you had your first period?**
    1. **Probe**: How did he or she react?
    2. **Probe**: What did that they say?
12. **Tell me about some of the cultural beliefs and practices around menstruation?**
    1. **Probe:** What are your thoughts?
    2. **Probe:** Did these beliefs and practices apply to you?. If so how did you feel about it?
13. **What other changes did you notice in your body when growing up?**
    1. **Probe**: What were your thoughts?
    2. **Probe**: How did you feel?
    3. **Probe**: What was your parent’s reaction?
    4. **Probe**: How did your relatives react?
14. **Have you ever been informed about menstruation and body changes before reaching your first menstruation?**
    1. **Probe**: Where did you learn about it?
    2. **Probe**: What did they say/tell you?
    3. **Probe**: When did you start thinking about opposite sex?
15. **What is some advice you might like to give young girls to prepare them for their periods?**
16. **Do you have further questions or comments?**

**CONCLUSION AND CLOSURE:**

We have now come to the end of our discussion. I would like to sincerely thank you for your participation and contribution. Be reminded again that your comments will remain confidential and your name will not be attached to any comments you have made.

Thank you.
